# Solvent-free thallium(I) tetra­kis­[3,5-bis­(tri­fluoro­meth­yl)phen­yl]borate: crystal structure, supra­molecular inter­actions and anisotropic thermal expansion

**DOI:** 10.1107/S2053229626008454

**Published:** 2026-09-05

**Authors:** Johannes Stephan, Lena Schröck, E. Eja Petersen, Romy L. Ettlinger, Alexander Pöthig

**Affiliations:** a Catalysis Research Center, Technical University of Munich, Ernst-Otto-Fischer-Straße 1, Garching, 85748, Germany; b Chair of Inorganic and Metal-Organic Chemistry, TUM School of Natural Sciences, Technical University of Munich, Lichtenbergstraße 4, Garching, 85748, Germany; Cinvestav, Mexico

**Keywords:** crystal structure, thallium(I) salts, weakly coordinating anion, supra­molecular inter­actions, anisotropic thermal expansion

## Abstract

Thallium salts of weakly coordinating anions are common reagents in organometallic chemistry for generating reactive cationic species *via* salt metathesis. Despite their frequent use, their com­position is often ambiguous due to cocrystallized solvents, rendering well-characterized solvent-free structures rare. Herein, we report the synthesis, crystal structure and thermal properties of truly solvent-free thallium(I) tetra­kis­[3,5-bis­(tri­fluoro­meth­yl)phen­yl]borate, Tl[BC_32_H_12_F_24_], as determined by single-crystal X-ray diffraction (SC-XRD), powder X-ray diffraction (PXRD) and thermogravimetric analysis (TGA). The structure features short Tl⋯F contacts and a com­plex supra­molecular architecture that is closely related to disorder and tilting of the anion arene rings, which generate distinct inter­molecular inter­action motifs. Key features include a herringbone arrangement of nonclassical C—H⋯F hy­dro­gen bonds and a ‘fluorous’ inter­layer defined by F⋯F and C—F⋯π contacts. This leads to hinge-like networks in the structure, depending on the tilt of the arene rings. These features are associated with anisotropic thermal expansion, with significantly greater expansion along the crystallographic *a* and *b* axes, as inferred from PXRD measurements. In agreement with the solvent-free structure determined by SC-XRD, TGA confirms that the bulk material is solvent-free.

## Abstract 2

The solvent-free structure of the thallium(I) salt of the weakly coordinating anion tetra­kis­[3,5-bis­(tri­fluoro­meth­yl)phen­yl]borate, Tl[BC_32_H_12_F_24_], exhibits short Tl⋯F contacts and a supra­molecular architecture governed by C—H⋯F hy­dro­gen bonds and weak F⋯F and C—F⋯π inter­actions. Disorder and tilting of the arene rings generate different close-contact motifs, giving rise to hinge-like inter­molecular inter­actions and pronounced anisotropic thermal expansion along the crystallographic *a* and *b* axes.

## Introduction

Owing to their high chemical stability and their low nucleophilicity, weakly coordinating anions (WCAs) have significantly influenced coordination chemistry over the last three decades (Krossing & Raabe, 2004 ▸; Riddlestone *et al.*, 2018 ▸). In particular, their ability to generate reactive cationic species without masking the cation’s reactivity through undesirable coordination has made them valuable building blocks for, *e.g.* catalysis, bond-activation reactions and electrochemistry. Prime examples of reactive cationic com­pounds, stabilized by WCAs, include the silylium ion (Kim *et al.*, 2002 ▸), the nonclassical 2-norbornyl carbocation (Scholz *et al.*, 2013 ▸), as well as metals in uncommon environments or oxidation states, such as metal–alkane com­plexes (Evans *et al.*, 1997 ▸; Pike *et al.*, 2012 ▸) or an Au^II^ centre with unusual ligands (Seidel & Seppelt, 2000 ▸). Among the numerous WCAs reported to date (Reed, 1998 ▸; Krossing, 2001 ▸; Kelling *et al.*, 2024 ▸; He *et al.*, 2025 ▸), the tetra­aryl­borate anions tetra­kis­[3,5-bis­(tri­fluoro­meth­yl)phen­yl]borate (BAr^F^) (Nishida *et al.*, 1984 ▸) and tetra­kis­(penta­fluoro­phen­yl)borate, [B(C_6_F_5_)_4_]^−^ (Massey & Park, 1964 ▸; Lambert & Zhang, 1993 ▸), represent some of the most frequently employed representatives, owing to their excellent weakly coordinating properties and straightforward preparation.

WCAs are typically introduced *via* salt metathesis or halide abstraction reactions, for which application silver and thallium salts have proven particularly useful reagents (Heintz *et al.*, 2002 ▸). Their utility arises from the favourable combination of their high solubility in organic solvents and their propensity to form poorly soluble halide salts. Although thallium salts are substanti­ally more toxic than their silver counterparts, they are generally less oxidizing and therefore more redox-innocent reagents. Consequently, thallium WCA salts may provide access to low-valent metal com­plexes that are difficult to obtain using silver salts, which may otherwise induce undesired oxidation processes (Salem *et al.*, 2008 ▸).

Despite their widespread use in synthetic inorganic and organometallic chemistry (Heintz *et al.*, 2002 ▸; Alberti & Pörschke, 2004 ▸), crystal structures of silver and thallium WCA salts remain com­paratively scarce, particularly in the absence of cocrystallized solvent mol­ecules. The weak inter­actions between the cation and the anion often permit additional stabilization through weak solvent coordination, making solvate formation a common feature of these com­pounds (McSkimming, 2025 ▸; Carreras *et al.*, 2017 ▸). As a result, structures of genuinely solvent-free WCA salts are relatively rare, which hampers insight into the intrinsic cation–anion inter­actions that govern their solid-state structures. Concerning in particular thallium tetra­aryl­borate salts, a solvent-free modification has so far been reported only for Tl[B(C_6_F_5_)_4_] (Parvez *et al.*, 2005 ▸). ‘Tl(BAr^F^)’, in contrast, has previously been described exclusively as a di­chloro­methane solvate containing 0.33 mol­ecules of di­chloro­methane per formula unit of Tl(BAr^F^) (Hughes *et al.*, 1997 ▸).
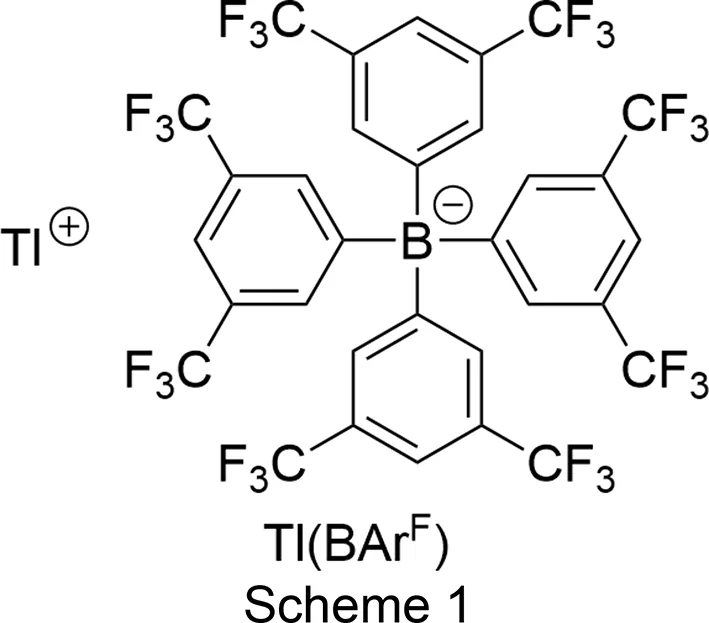


In this article, we report the crystal structure and bulk properties of truly solvent-free thallium(I) tetra­kis­[3,5-bis­(tri­fluoro­meth­yl)phen­yl]borate [Tl(BAr^F^)]. Single-crystal X-ray diffraction (SC-XRD) reveals the solid-state structure of the unsolvated salt and allows a detailed assessment of the weak Tl⋯F inter­actions that dominate its crystal packing, amongst weak F⋯F and C—H⋯F inter­actions. Phase purity and bulk crystallinity were confirmed by powder X-ray diffraction (PXRD), while thermogravimetric analysis (TGA) was used to confirm the solvent-free nature of the bulk material and elucidate its thermal stability.

## Experimental

### Synthesis and crystallization

Thallium tetra­kis­[3,5-bis­(tri­fluoro­meth­yl)phen­yl]borate was synthesized based on established procedures (Hughes *et al.*, 1997 ▸; Alberti & Pörschke, 2004 ▸; Stephan *et al.*, 2025 ▸). This in­volves protolysis of thallium(I) ethoxide with H(Et_2_O)(BAr^F^) (Brookhart’s acid). Following the most recent protocol (Stephan *et al.*, 2025 ▸), thallium(I) ethoxide (0.2604 g, 1.04 mmol, 1.06 equiv.) was added to a solution of an equimolar amount of Brookhart’s acid (1.0032 g, 0.98 mmol, 1.00 equiv.) in dry diethyl ether (5 ml) in a Schlenk tube under an argon atmosphere. After stirring for 10 min at room tem­per­a­ture, all volatiles were removed *in vacuo* to yield a beige powder. Immediately after adding thallium ethoxide, the solution may turn cloudy, presumably because thallium chloride may form from traces of sodium chloride (<1%) in H(Et_2_O)(BAr^F^). The TlCl precipitate can be easily removed by filtration using a syringe filter inside a glovebox. Redissolving the crude Tl(BAr^F^) in dry fluoro­benzene (4 ml) and layering the solution with dry *n*-hexane (10 ml) reproducibly yields colourless crystals several millimetre in size at room tem­per­a­ture after 2 to 3 d, with a total yield of 75–85%. Alternatively, smaller-sized crystals can easily be obtained by cooling a Tl(BAr^F^) solution in a 1:1 mixture (8 ml) of fluoro­benzene and *n*-hexane to −32 °C overnight with identical yield and properties.

For SC-XRD analysis, Tl(BAr^F^) single crystals were selected in an argon-filled glovebox with appropriate dust filters (to prevent atmospheric contamination with thallium) and covered in perfluorinated ether on a microscope slide inside the glovebox. A suitable single crystal was then mounted on a MicroMount Kapton micro­sampler, transferred to the dif­frac­tom­eter and frozen under a stream of cold nitro­gen (100 K).

### Refinement

Crystal data, data collection and structure refinement details are summarized in Table 1 ▸. Anharmonic motion of the Tl atom (Tl1) was refined using a fourth-order Gram–Charlier expansion as implemented in *olex2.refine* (Bourhis *et al.*, 2015 ▸) within *OLEX2* (Dolomanov *et al.*, 2009 ▸). H atoms were located in difference Fourier maps, but were calculated in ideal positions using a riding model, with C—H = 0.99 Å and *U*_iso_(H) = 1.2*U*_eq_(C). The disorder of the arene rings of the BAr^F^ anions over three positions, tilted by 5.49 and 6.15°, was modelled using a split-layer refinement, and the geometry of the second and third component with minor occupancy were restrained to be the same as in PART 1. To ensure convergence, restraints were used within reasonable limits. Additionally, the C—F bond lengths for the CF_3_ groups of the parts with minor occupancy were restrained to 1.34 (2) Å. The carbon frameworks of the two parts with minor occupancies, except for the F atoms, were modelled as rigid bodies. Thermal displacement parameters for certain F atoms were constrained to be equal to their counterparts in PART 1, as well as the boron-bound C atoms, which were constrained to be equal to C1 (PART 1).

### Powder X-ray diffraction

Powder X-ray diffraction data were acquired using sealed borosilicate glass capillaries with a diameter of 0.5 mm, filled under an argon atmosphere in a glovebox with appropriate dust filters. Intensities were collected at room tem­per­a­ture on a STOE Stadi P two-circle powder X-ray diffractometer with Debye–Scherrer geometry. The diffractometer was equipped with a sealed-tube X-ray source emitting Mo *K*α radiation, a curved Ge monochromator and a Dectris Mythen2 DCS4 detector. Cell indexing and refinement were performed using *WinXPOW* (Version 3.05; STOE & Cie GmbH, Darmstadt, Germany).

### Thermogravimetric analysis

Thermogravimetric analysis (TGA) was performed on a Mettler Toledo TGA/DSC3+ under a constant flow of argon (25 ml min^−1^). A sample of Tl(BAr^F^) was ground in an agate mortar in an argon-filled glovebox and approximately 2 mg were weighed into a calcined alumina crucible, which was placed into the TGA device. Heating was applied in a range between 30 and 1000 °C, with a heating rate of 2 °C min^−1^.

## Results and discussion

Solvent-free Tl(BAr^F^) crystallizes in a primitive tetra­gonal space group, as derived from SC-XRD. Due to the weakly coordinating nature of the fluoro­benzene solvent that was used for crystallization, a structure containing no solvent mol­ecules was obtained. This is supported by PXRD (see Fig. S8 in the supporting information), which shows the same solvent-free unit cell for the bulk material, elemental analysis (see Table S1) and ^1^H NMR spectrum in *d*_3_-aceto­nitrile solution (see Fig. S1). The ^1^H NMR spectrum shows only two closely adjacent peaks at 7.70 and 7.67 ppm, which can be attributed to the protons of the BAr^F^ anion.

The solvent-free crystal structure of Tl(BAr^F^), as determined by SC-XRD, was modelled in the centrosymmetric tetragonal space group *P*4/*n* (No. 85, *Z* = 2) with two formula units per unit cell. In fact, the structure appears to exhibit pronounced pseudocentrosymmetry. Initially, space group determination using *XPREP* (Bruker, 2014 ▸) suggested the centrosymmetric space groups *P*4/*n* and *P*4/*nmm*, whereas intensity statistics with |*E*^2^ – 1| = 0.689 were indicative of a noncentrosymmetric or chiral space group. Structure solution using *SHELXT* (Sheldrick, 2015*a* ▸) was successful only for the centrosymmetric space group *P*4/*n* and the Sohncke space group *P*4. We therefore tested refinement for both solutions and the *P*4/*n* structure model proved more accurate. Briefly, refinement in *P*4 converged satisfactorily as a two-com­ponent inversion twin with a final BASF ≃ 0.5, but yielded only slightly lower *R* values. However, the Flack (1983 ▸) parameter was refined to a statistically significant value of 0.50 (3), indicating inversion ambiguity consistent with a centrosymmetric model, and the *PLATON* ADDSYM analysis (Spek, 2020 ▸) also recommended the higher-symmetry space group *P*4/*n*. The structure model in *P*4/*n* (instead of *P*4) additionally yielded physically more reasonable atomic displacement parameters, especially for the light elements boron and carbon. Therefore, the centrosymmetric model was adopted as the more accurate description of the structure.

Concomitantly, anharmonic motion was refined for the Tl atom using *olex2.refine* (Bourhis *et al.*, 2015 ▸), as implemented in the *OLEX2* software package (Dolomanov *et al.*, 2009 ▸). Fourth-order anharmonic refinement of the Tl atom resulted in a substanti­ally improved description of the structure, as evidenced by the conventional residual indices decreasing from *R*1 = 3.45% and *wR*2 = 9.08% for the harmonic model to *R*1 = 2.06% and *wR*2 = 5.26% for the anharmonic model (see Table 1 ▸). In addition, high residual electron density of 7.89 e Å^−3^ in the vicinity of the Tl atom (<1 Å) was virtually com­pletely removed to 0.57 e Å^−3^ in the anharmonic model. Although the resolution criterion proposed by Kuhs (1988 ▸), which recommends a minimum resolution of 0.58 Å for reliable fourth-order anharmonic refinement, is not fulfilled with 0.66 Å, the anharmonic model appears physically meaningful. The resulting probability density function remained positive throughout the inspected region (see Fig. S7), and only a limited number of Gram–Charlier coefficients were found to be statistically significant. The expansion is dominated by the third-order C333 coefficient [−4.11 (8) × 10^−6^; 51.4σ], while for the fourth-order terms, D3333 [4.20 (11) × 10^−7^; 38.2σ], represents the dominant contribution. All remaining coefficients are either symmetry-restricted, statistically insignificant or (marginally) significant at the 10σ level. For this reason, the anharmonic model was adopted as a valid and more realistic representation of Tl atomic motion. More details on refinement and the Gram–Charlier coefficients can be found in the supporting information (see Tables S2 and S3).

The asymmetric unit of Tl(BAr^F^) com­prises one-quarter of both the thallium cation and the BAr^F^ anion (see Fig. 1 ▸). The Tl atom lies directly on two of the fourfold axes of the unit cell, which also represent the axes along which anharmonic motion occurs (see Fig. S7). Additionally, the B atoms occupy the two remaining *C*_4_ axes. Therefore, the BAr^F^ anion exhibits *S*_4_ symmetry. The tetra­hedral coordination environment of the B atoms is slightly distorted, as indicated by the C1—B1—C1^iv^ angle of 109.71 (5)° [symmetry code: (iv) *y* + 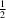, −*x* + 1, −*z* + 2], which deviates from the angle of 109.5° in an idealized tetra­hedron.

There are three distinct correlated positions of the arene rings, tilted by 5.49 and 6.15° (see Fig. S5), with the three com­ponents refining to occupancies of 0.579 (7), 0.263 (7) and 0.158 (5). This disorder is directly connected to a supra­molecular feature of the structure, giving rise to two domains in which different inter­molecular fluorine-based inter­actions dominate (see Fig. 2 ▸ and Table 2 ▸). The major com­ponent (PART 1) is associated with a network of weak nonclassical C—H⋯F hy­dro­gen bonds, whereas the minor com­ponents (PART 2 and PART 3) are characterized by short F⋯F inter­actions and weak C—F⋯π inter­actions. Generally, the co-existence of both nonclassical C—H⋯F hy­dro­gen bonds and F⋯F inter­actions with dispersive character within the same structure, while simultaneously leading to self-sorting, is not uncommon in fluorine-rich organic mol­ecules (Pickl *et al.*, 2024 ▸). Overall, the structure of Tl(BAr^F^) displays hinge-like supra­molecular inter­actions between the BAr^F^ anions, held together by com­paratively weak inter­molecular inter­actions. In the part with major occupancy, a weak but directional C—H⋯F inter­action between H6 and F1^ii^ (Table 2 ▸) is pres­ent, suggesting a nonclassical hy­dro­gen bond, with the H6⋯F1^ii^ distance of 2.621 (7) Å being slightly shorter than the sum of the van der Waals radii. Due to the *S*_4_ symmetrical nature of the BAr^F^ anion, this leads to a herringbone-type arrangement of C—H⋯F inter­actions, which produces layers along the *ab* plane. A similar network of nonclassical hy­dro­gen bonds is missing in the two minor-occupancy regions, as deduced from the corresponding H6*A*⋯F1*A*^ii^ [2.868 (14) Å] and H6*B*⋯F1*B*^ii^ [2.856 (17) Å] distances. Instead, there are short but weak C—F⋯π contacts (see Table 2 ▸) between F atoms and adjacent aromatic rings. The geometry, with ap­proxi­mately perpendicular ring planes and an η^2^-like inter­action motif, suggests weak but directed C—F⋯π inter­actions. Specifically for PART 2, there is also a short F3*A*⋯F4*A*^i^ contact (Table 2 ▸) of 2.90 (3) Å between the CF_3_ groups of the anions. In the two parts with minor occupancy, these short C—F⋯π and F⋯F contacts form a ‘fluorous’ inter­layer around the Tl atoms; the interlayer lies parallel to the *a* and *b* axes, but perpendicular to the *c* axis. Therefore, the rings of the BAr^F^ anions either twist against each other in the part with major occupancy to form nonclassical hy­dro­gen-bond networks, or the rings tilt towards each other in the two parts with minor occupancy to form an inter­layer of short F⋯F, as well as weak η^2^-like C—F⋯π contacts (see Fig. S6). This structural flexibility is somewhat reminiscent of the ‘breathing’ described for soft crystalline materials (Horike *et al.*, 2009 ▸), although in the pre­sent­ed structure, no discrete open and closed states at the microscale or phase transitions at the macroscale are observed.

Besides supra­molecular networks and layered structures, close Tl⋯F contacts within the range 2.824 (16)–3.233 (9) Å were detected (see Table 3 ▸), rendering Tl(BAr^F^) a contact ion pair. Such contacts have also been reported for other thallium salts of weakly coordinating anions (Hughes *et al.*, 1997 ▸; Parvez *et al.*, 2005 ▸). The distances described in this work are among the shortest yet. In Tl(BAr^F^)·0.33CH_2_Cl_2_, the Tl⋯F distances within the sum of the van der Waals radii are 3.05 (6) and 3.18 (6) Å (Hughes *et al.*, 1997 ▸), while the distances in Tl[B(C_6_F_5_)_4_] lie between 2.942 (4) and 3.663 (4) Å (Parvez *et al.*, 2005 ▸). For com­parison, in thallium(I) fluoride, the inter­atomic Tl—F distances within the sum of the van der Waals radii (including actual covalent bonds) range from 2.251 (17) to 3.496 (14) Å (Alcock & Jenkins, 1974 ▸). Despite such close contacts in solvent-free Tl(BAr^F^), the C—F bond lengths seem to be largely unaffected, since they are in a reasonable range between 1.335 (4) and 1.366 (7) Å for the part with major occupancy, for which the C—F distances were allowed to refine freely. Overall, each Tl atom is surrounded by 12 F atoms, resulting in a cubocta­hedral pseudo-coordination polyhedron. The coordination environment is strongly dis­torted, as indicated by the differing range of Tl⋯F contact distances between 2.824 (16) and 3.233 (9) Å, as well as the view along the crystallographic *b* axis (see Fig. 3 ▸). This is a con­sequence of the CF_3_ moieties of the BAr^F^ anions inter­acting with the Tl atom, either with two F atoms or only one at the basis of the cubocta­hedron.

Hirshfeld surface analysis of Tl1, using *CrystalExplorer* (Spackman *et al.*, 2021 ▸), confirms Tl⋯F contacts as the sole inter­actions for the Tl atoms with the surrounding anions. The Hirshfeld surface mapped over *d*_norm_ for the part with major occupancy revealed several short Tl⋯F contacts as bright red spots in the graphical representation [see Fig. 4 ▸(*a*)]. The two-dimensional fingerprint plot of *d*_e_ over *d*_i_ [the distances to the nearest atom inter­nal or external to the surface; Fig. 4 ▸(*b*)] shows that these contacts are indeed the only inter­actions between the thallium cations and the BAr^F^ anions, accounting for 100% of the total surface area.

Tl⋯F contacts, forming a distorted coordination environment around Tl, also affect the packing of Tl(BAr^F^). Alternating units of Tl⋯F cubocta­hedra lead to a zigzag motif of thallium cations, which is clearly visible along the crystallographic *a* axis [see Fig. 5 ▸(*a*)]. In the expanded unit cell, eight BAr^F^ anions surround one Tl atom, which results in a cage-like nearly cubic arrangement of BAr^F^ anions, with isolated Tl centres showing no Tl⋯Tl contacts [see Fig. 5 ▸(*b*)]. This matches well with previous reports on alkali-metal BAr^F^ salts, such as anhydrous and solvent-free Na(BAr^F^) (CCDC 1886446) and K(BAr^F^) (CCDC 1886447) (Martínez-Martínez & Weller, 2019 ▸), which also exhibit a virtually cubic arrangement of the weakly coordinating anions. The effective pseudo-coordination number in these salts is 8, whereas it is 12 in Tl(BAr^F^), most probably due to the larger radius of the Tl^I^ cation.

To confirm that the bulk of Tl(BAr^F^) has the same (solvent-free) structure as derived from SC-XRD, we performed PXRD measurements of the obtained material. The diffractogram, acquired at room tem­per­a­ture rather than at 100 K as in the SC-XRD measurement, matches well with its SC-XRD counterpart (see Figs. 6 ▸ and S8). The indexed unit cell exhibits primitive tetra­gonal symmetry, suggesting a largely similar structure to that at 100 K. Inter­estingly, com­paring the unit cells at 100 K and at room tem­per­a­ture reveals anisotropic elongation along the crystallographic *a* and *b* axes. Both axes expand to 13.859 (7) Å, which corresponds to an increase by 2.7%. In contrast, the *c* axis remains nearly unchanged with a length of 9.675 (7) Å, which corresponds to a com­parably small expansion by 0.5%. This is also reflected in the overlay of the low-tem­per­a­ture structure with the simulated room-tem­per­a­ture structure [see Fig. 6 ▸(*b*)]. The view along the *b* axis shows virtually no expansion for the unit cell along the *c* axis but pronounced expansion along the *a* and *b* axes, which is also clearly visible in the view along the *c*-axes of the overlay. This thermal expansion behaviour may tentatively correlate with the supra­molecular networks that produce layer-like structures in both com­ponents of the disorder. In the part with major occupancy, the herringbone arrangement of nonclassical hy­dro­gen bonds forms layers along the *ab* plane, which are perpendicular to the *c* axis. In the minor parts of the structure, short C—F⋯π and F⋯F contacts form a ‘fluorous’ inter­layer around the Tl atoms, which also lies parallel to the *a* and *b* axes, but perpendicular to the *c* axis. While these two types of layers lie in the same direction within the unit cell and can ‘glide’ com­parably easily against each other along the *a* and *b* axes, we assume that the *c* axis appears to be more ‘locked’, with the networks perpendicular to it. This presumably reduces flexibility along the *c* axis and allows for easier movement of the structure along the *a* and *b* axes.

In addition, TGA of the obtained Tl(BAr^F^) under an argon atmosphere (see Fig. 7 ▸) shows that the bulk material is solvent-free, as anti­cipated from the SC-XRD structure. No mass loss is observed until an onset tem­per­a­ture of 245 °C, which marks the decom­position point of Tl(BAr^F^). At its first decom­position step, the sample of Tl(BAr^F^) loses 59.46% of weight, which corresponds to the loss of the borane B[C_6_H_3_(CF_3_)_2_]_3_ (*M* = 650.12 g mol^−1^; 60.90%) from the BAr^F^ borate anion. The peak tem­per­a­ture of this decom­position is 266 °C, as determined from the derivative thermogravimetry (DTG) plot, *i.e.* the first derivative of the TGA curve. The presumably remaining TlC_6_H_3_(CF_3_)_2_ species decom­poses over a wider tem­per­a­ture range, eventually converging to a residual mass of 0 mg. This is most likely due to the formation of volatile TlF, which has a boiling point of 655 °C (Perry & Phillips, 1995 ▸), causing it to evaporate under these conditions. Similar thermal properties and decom­position tem­per­a­tures have also been reported for systems containing structurally related borate anions, such as [B(C_6_F_5_)_4_]^−^ in imidazolium-based structures (Zhang *et al.*, 2012 ▸) or Na[B{C_6_H_3_(SF_5_)_2_}_4_] (Langford *et al.*, 2019 ▸).

## Conclusion

In summary, the crystal structure of truly solvent-free Tl(BAr^F^) is pre­sent­ed. The structure com­prises among the shortest Tl⋯F contacts reported to date, which represent the primary direct inter­action between the thallium cation and the BAr^F^ anion. The com­pound exhibits a com­plex supra­mole­cular organization characterized by hinge-like anion–anion inter­actions that are closely associated with disorder of the aryl rings. This gives rise to a herringbone arrangement of nonclassical C—H⋯F hy­dro­gen bonds in the major disorder com­ponent, while the minor com­ponents form ‘fluorous’ inter­layers dominated by C—F⋯π and F⋯F contacts. These supra­molecular features correlate with anisotropic thermal ex­pansion of the unit-cell parameters along the *a* and *b* axes, as determined by variable-tem­per­a­ture PXRD measurements. In addition, PXRD and TGA analyses confirm that the bulk material is phase-pure and retains the solvent-free structure observed by SC-XRD.

## Supplementary Material

Crystal structure: contains datablock(s) I, global. DOI: 10.1107/S2053229626008454/zo3075sup1.cif

Structure factors: contains datablock(s) I. DOI: 10.1107/S2053229626008454/zo3075Isup2.hkl

Supporting information file. DOI: 10.1107/S2053229626008454/zo3075Isup3.cml

Data for cell determination using PXRD. DOI: 10.1107/S2053229626008454/zo3075sup4.txt

Data for unit-cell indexing (PXRD). DOI: 10.1107/S2053229626008454/zo3075sup5.txt

Elemental analysis, NMR spectra, additional SC-XRD data and PXRD data. DOI: 10.1107/S2053229626008454/zo3075sup6.pdf

CCDC reference: 2581457

## Figures and Tables

**Figure 1:**
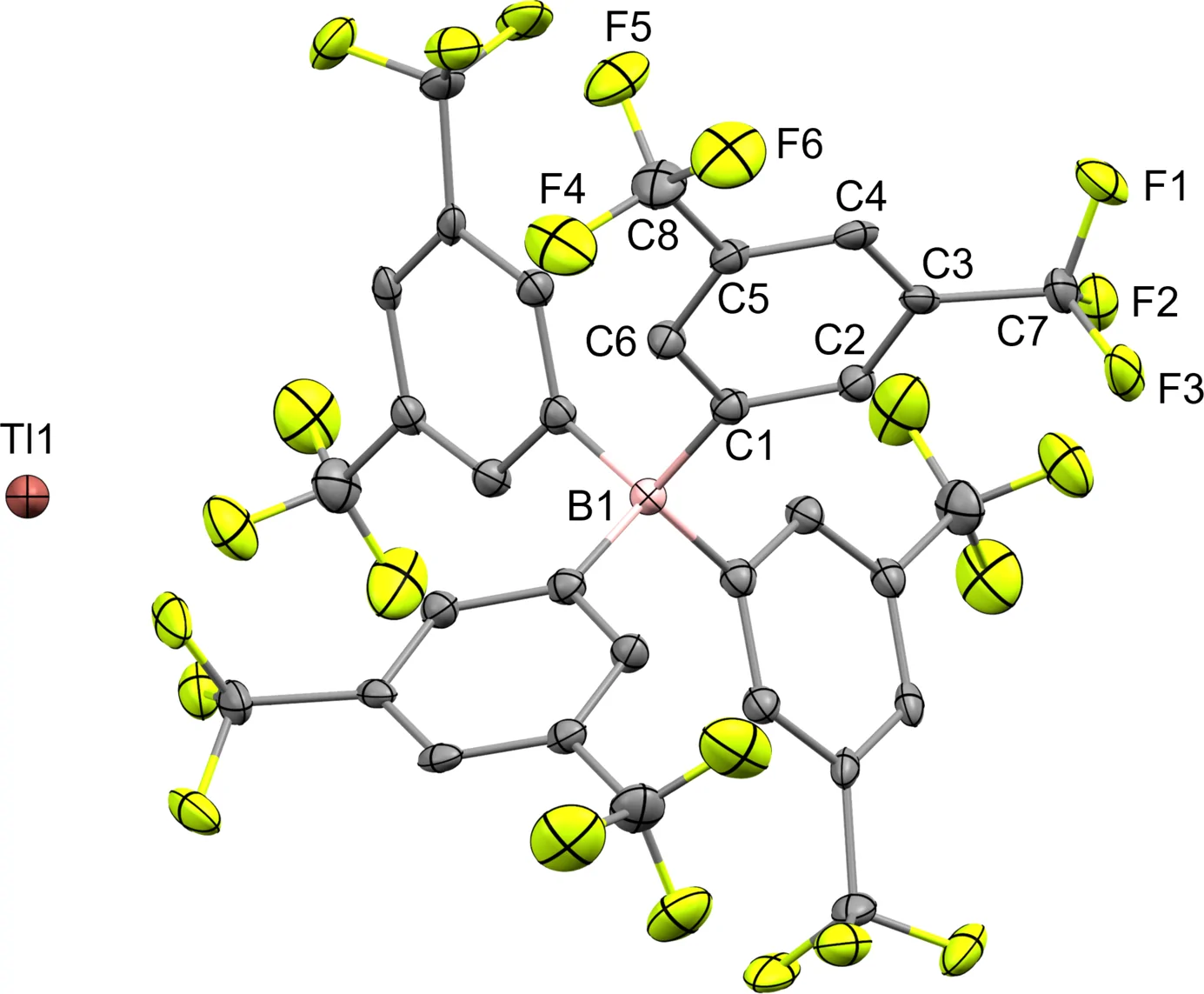
The structure of Tl(BAr^F^), as determined by SC-XRD, with the atom-numbering scheme, viewed along the *c* axis. For clarity, the figure only shows PART 1, with only the atoms of the asymmetric unit labelled and H atoms omitted. Atoms H2, H4 and H6 are attached to the C atoms with the same numbers. All displacement ellipsoids are drawn at the 50% probability level.

**Figure 2:**
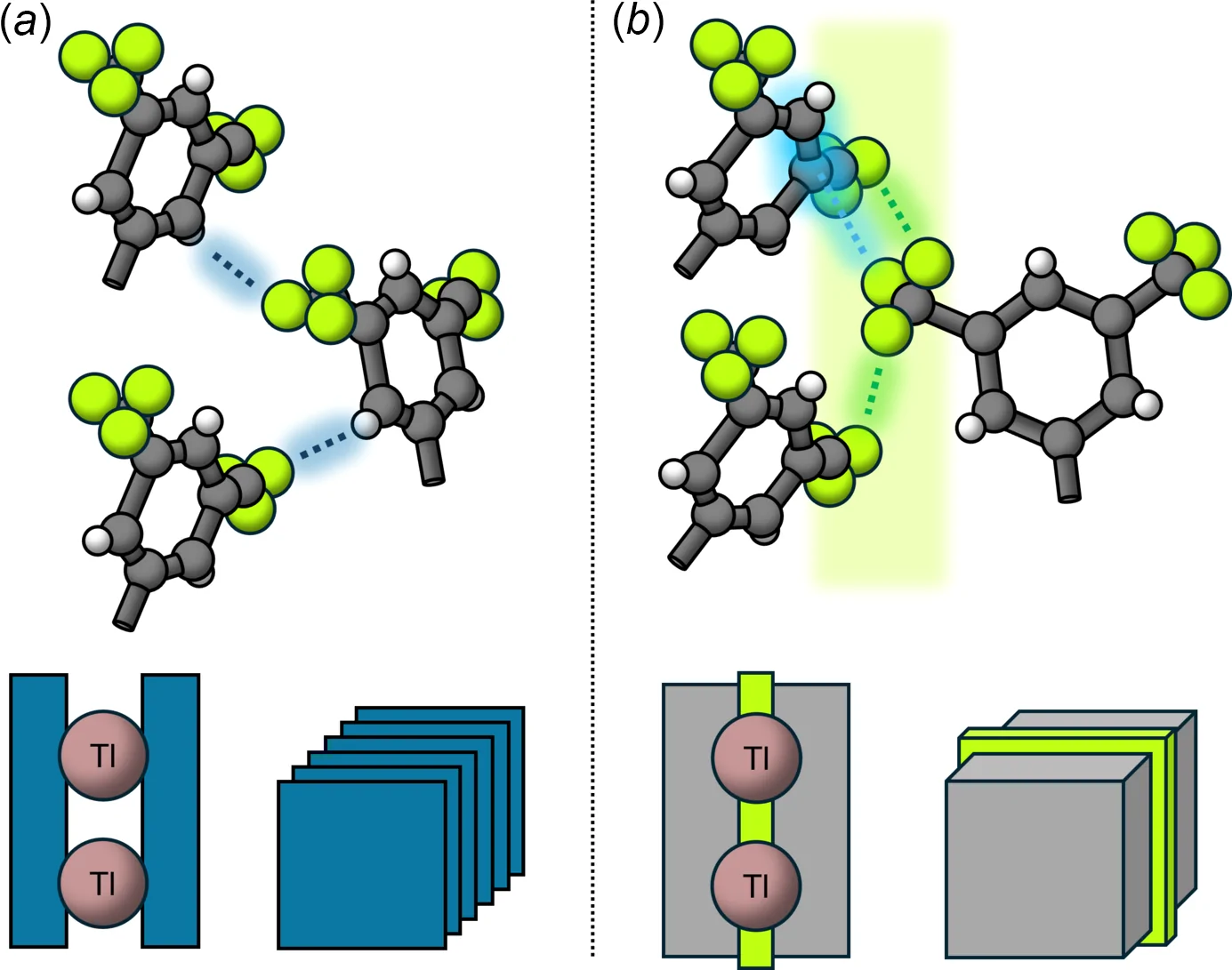
Schematic representation of the hinge-like inter­actions between the BAr^F^ anions. (*a*) In the part with major occupancy, layers of nonclassical hy­dro­gen bonds form a herringbone pattern along the *a* and *b* axes, and a stacking motif along the *c* axis, with the thallium ions in between (bottom left). (*b*) C—F⋯π and F⋯F contacts in the minor com­ponents form ‘fluorous’ inter­layers along the *a* and *b* axes and a barrier-like layer along the *c* axis (bottom right).

**Figure 3:**
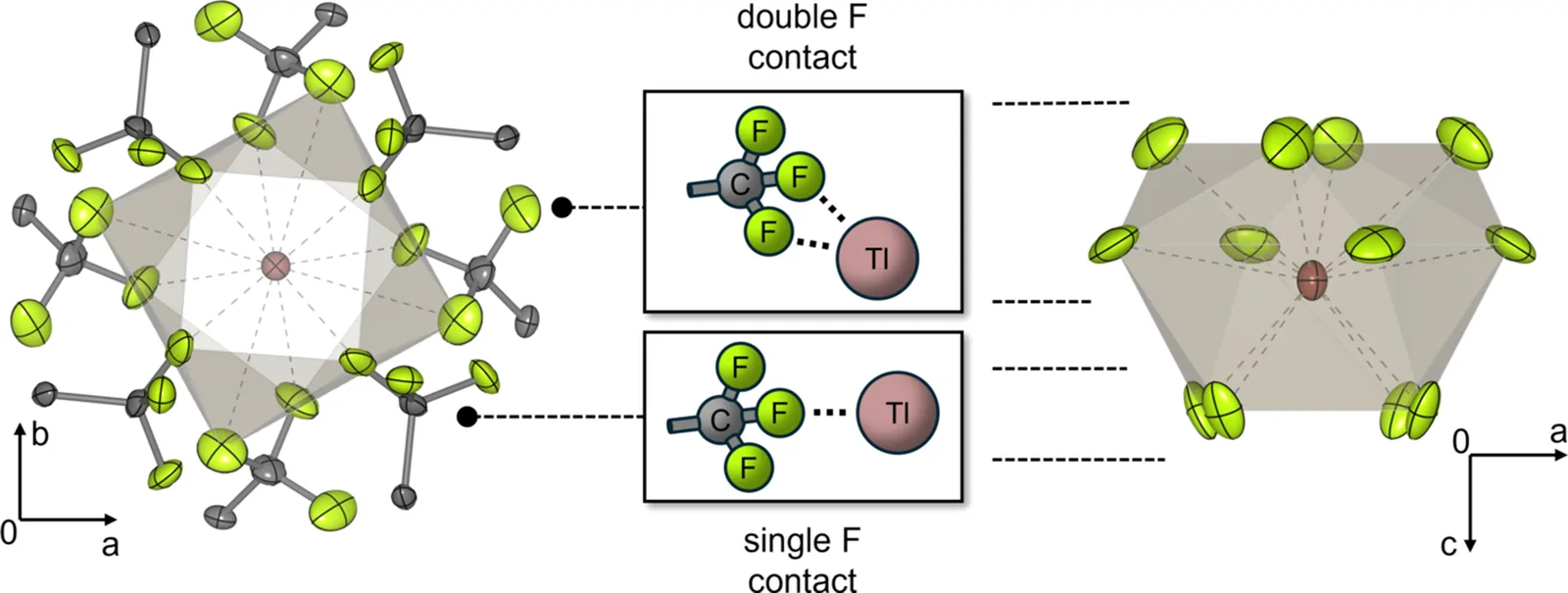
Distorted cubocta­hedral pseudo-coordination polyhedron around the central Tl atom, either viewed along the *c* axis (left) or the *b* axis (right), with displacement ellipsoids drawn at the 50% probability level. The CF_3_ groups inter­act with the Tl atom *via* either two F atoms as a double contact or *via* one F atom as a single contact.

**Figure 4:**
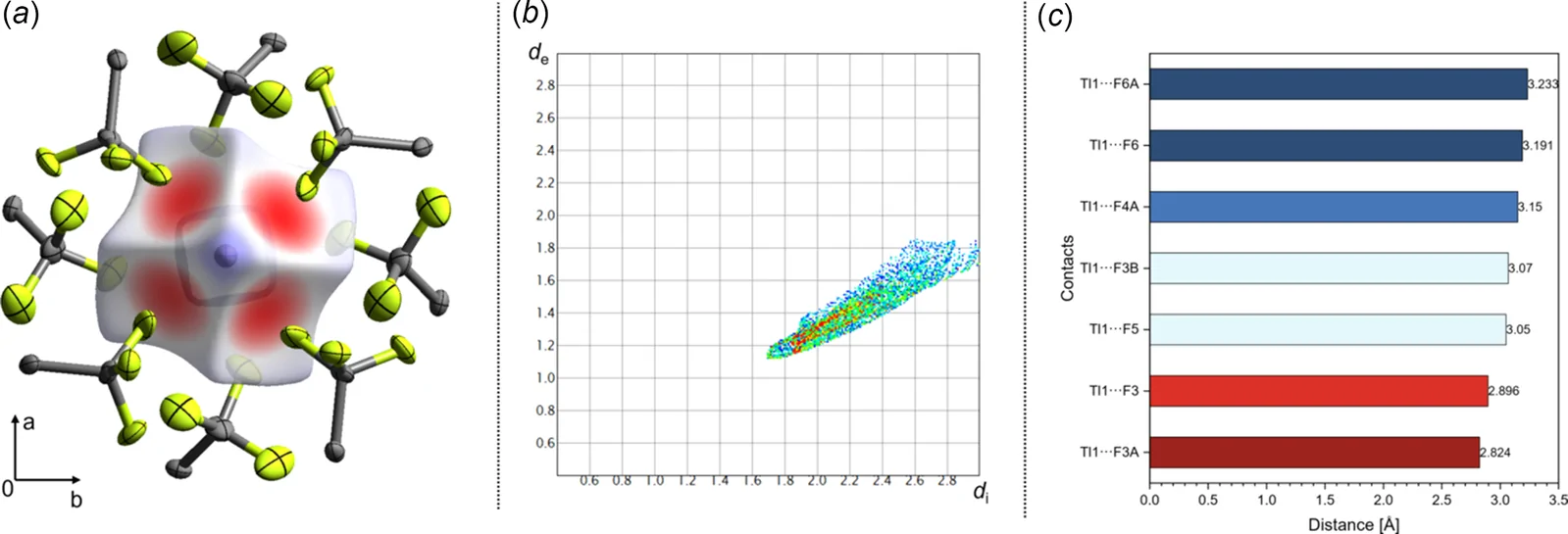
(*a*) Hirshfeld surface of Tl1, generated with *CrystalExplorer*, indicating short Tl⋯F contacts as bright red spots on the surface representation, as viewed along the *c* axis. (*b*) The fingerprint plot of *d*_e_ over *d*_i_ shows only Tl⋯F inter­actions. (*c*) Histogram plot of the differing Tl⋯F contact distances, revealing Tl1⋯F3*A* and Tl1⋯F3 as the shortest contacts (red bars).

**Figure 5:**
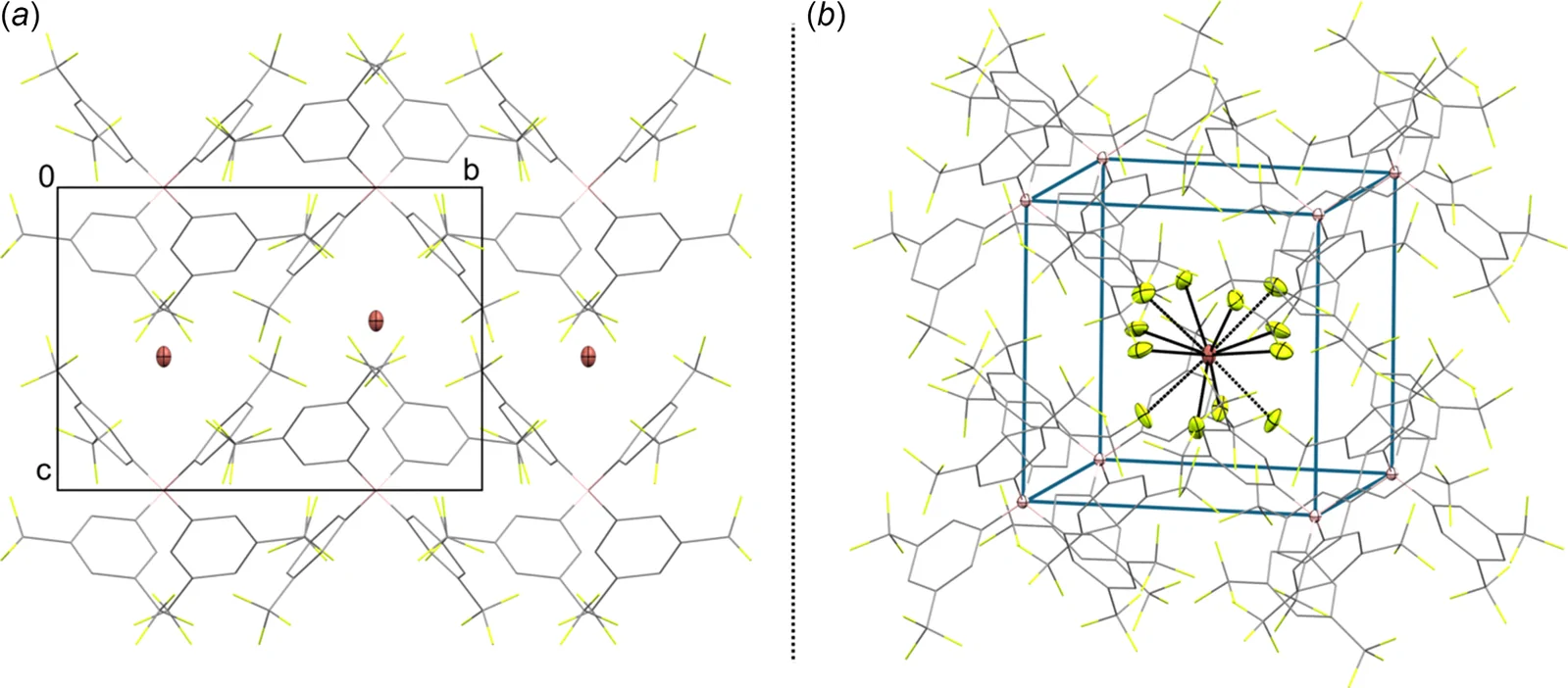
(*a*) Packing diagram of Tl(BAr^F^), viewed along the *a* axis, showing a zigzag alignment of Tl atoms. (*b*) The expanded unit cell exhibits a virtually cubic arrangement of eight BAr^F^ anions around the central Tl atom. Tl atoms, as well as B atoms and short-contact F atoms in part (*b*), are displayed as displacement ellipsoids at the 50% probability level; the remaining atoms are displayed in wireframe mode.

**Figure 6:**
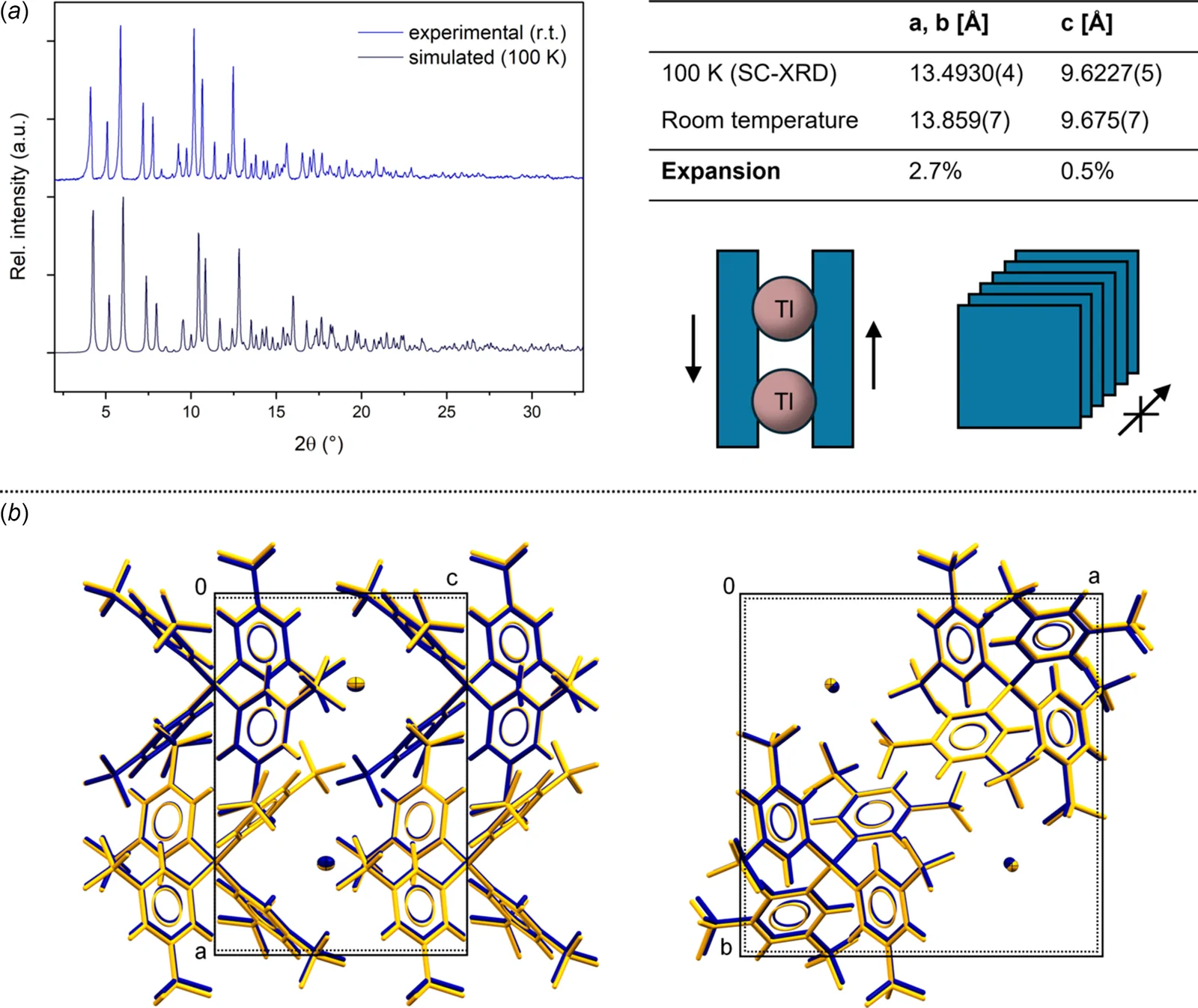
(*a*) Comparison of the powder X-ray diffractogram of Tl(BAr^F^) recorded at room tem­per­a­ture (top left trace, blue) and its simulated diffractogram based on the SC-XRD structure at 100 K (bottom left trace, dark blue). Anisotropic thermal expansion occurs along the *a* and *b* axes, with layers ‘gliding’ against each other along *a* and *b*, while the *c* axis does not expand significantly because both layer-like arrangements within the structure are perpendicular to it. (*b*) Overlay of the SC-XRD structure at 100 K (dark blue, dotted unit-cell box) and the simulated room-tem­per­a­ture structure (orange, solid unit-cell box), as viewed along the *b* axis (bottom left) and the *c* axis (bottom right).

**Figure 7:**
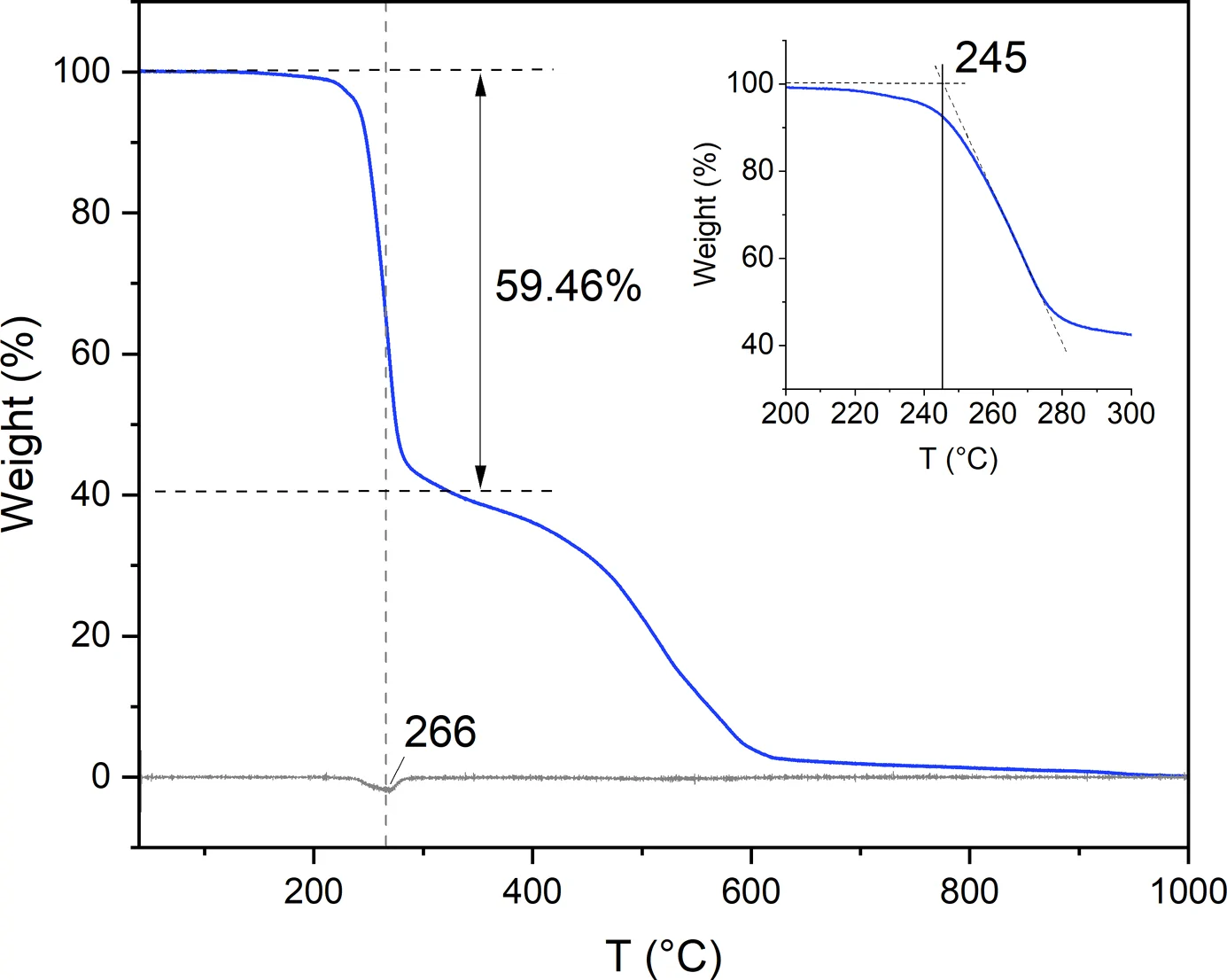
TGA (blue) and DTG (grey, bottom) curves of solvent-free Tl(BAr^F^) between 30 and 1000 °C at a heating rate of 2 °C min^−1^ under an argon atmosphere with a flow rate of 25 ml min^−1^. Decom­position occurs at 245 °C (see zoom-in), with a peak tem­per­a­ture of 266 °C.

**Table 1:** Experimental details

Crystal data	
Chemical formula	Tl^+^·C_32_H_12_BF_24_^−^
*M*_r_	1067.63
Crystal system, space group	Tetragonal, *P*4/*n*
Temperature (K)	100
*a*, *c* (Å)	13.4930 (4), 9.6227 (5)
*V* (Å^3^)	1751.92 (12)
*Z*	2
Radiation type	Mo *K*α
μ (mm^−1^)	4.76
Crystal size (mm)	0.16 × 0.15 × 0.10

Data collection	
Diffractometer	Bruker D8 VENTURE
Absorption correction	Multi-scan (*SADABS*; Bruker, 2016 ▸)
*T*_min_, *T*_max_	0.633, 0.747
No. of measured, independent and observed [*I* ≥ 2σ(*I*)] reflections	87174, 3197, 2970
*R*_int_	0.055
(sin θ/λ)_max_ (Å^−1^)	0.757

Refinement	
*R*[*F*^2^ > 2σ(*F*^2^)], *wR*(*F*^2^), *S*	0.021, 0.053, 1.06
No. of reflections	3197
No. of parameters	284
No. of restraints	697
H-atom treatment	H atoms treated by a mixture of independent and constrained refinement
Δρ_max_, Δρ_min_ (e Å^−3^)	0.59, −0.86

Computer programs: *APEX4* (Bruker, 2022 ▸), *SAINT* (Bruker, 2019 ▸), *SHELXT* (Sheldrick, 2015*a* ▸), *SHELXL* (Sheldrick, 2015*b* ▸), *ShelXle* (Hübschle *et al.*, 2011 ▸), *Mercury* (Macrae *et al.*, 2020 ▸), *PLATON* (Spek, 2020 ▸), *enCIFer* (Allen *et al.*, 2004 ▸) and *FinalCIF* (Kratzert, 2026 ▸), *OLEX2* (Dolomanov *et al.*, 2009 ▸), *olex2.refine* (Bourhis *et al.*, 2015 ▸) and *VESTA 3* (Momma & Izumi, 2011 ▸).

**Table 2:** Selected inter­atomic distances (Å), suggesting a nonclassical C—H⋯F hy­dro­gen bond, as well as weak F⋯F and C—F⋯π inter­actions

H6⋯F1^ii^	2.621 (7)	F5*A*⋯C5*A*^iii^	3.153 (8)
F3*A*⋯F4*A*^i^	2.90 (3)	F5*B*⋯C4*B*^iii^	3.195 (12)
F5*A*⋯C4*A*^iii^	3.180 (9)	F5*B*⋯C5*B*^iii^	3.182 (12)

Symmetry codes: (i) −*x* + 1, −*y* + 1, −*z* + 1; (ii) −*y* + 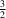, *x*, *z*; (iii) *y* + 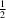, −*x* + 1, −*z* + 1.

**Table 3:** Close Tl⋯F contacts (Å), as identified by *PLATON* (Spek, 2020 ▸)

Tl1⋯F3	2.896 (7)	Tl1⋯F4*A*^i^	3.15 (2)
Tl1⋯F5^i^	3.050 (4)	Tl1⋯F6*A*^i^	3.233 (9)
Tl1⋯F6^i^	3.191 (12)	Tl1⋯F3*B*	3.070 (12)
Tl1⋯F3*A*	2.824 (16)	Tl1⋯F4*B*^i^	3.01 (3)

Symmetry code: (i) −*x* + 1, −*y* + 1, −*z* + 1.
