# Supplementary material for: Solvent-free thallium(I) tetra­kis­[3,5-bis­(tri­fluoro­meth­yl)phen­yl]borate: crystal structure, supra­molecular inter­actions and anisotropic thermal expansion
Source: Acta Crystallogr C Struct Chem. 2026 Sep 5;82(Pt 10):619–26. doi: 10.1107/S2053229626008454 (PMC13637802; doi:10.1107/S2053229626008454)
Supplement: Supplementary file 1 [file c-82-00619-sup6.pdf]

STRUCTURAL CHEMISTRY

Volume 82 (2026)

Supporting information for article:

Solvent-free thallium(I) tetrakis[3,5-bis(trifluoromethyl)phenyl]borate: crystal structure, supramolecular interactions and anisotropic thermal expansion

Johannes Stephan, Lena Schröck, E. Eja Petersen, Romy L. Ettlinger and Alexander Pöthig

S1. Elemental analysis and NMR data

Table S1 Elemental analysis of Tl(BAr^F) (Stephan *et al.*, 2025).

	Calculated	Found
C	36.00	35.70
H	1.13	1.09
N	-	-
S	-	-

Elemental analyses were conducted at the microanalytical laboratory at the Catalysis Research Center of the Technical University of Munich, using an Eurovector EA3000 CHNS combustion analyser. 1-2 mg of sample was weighed into a tin boat in a glovebox and sealed airtight, and then spontaneously combusted at 1800 °C. Combustion gases were separated by gas chromatography and detected with a thermal conductivity detector.

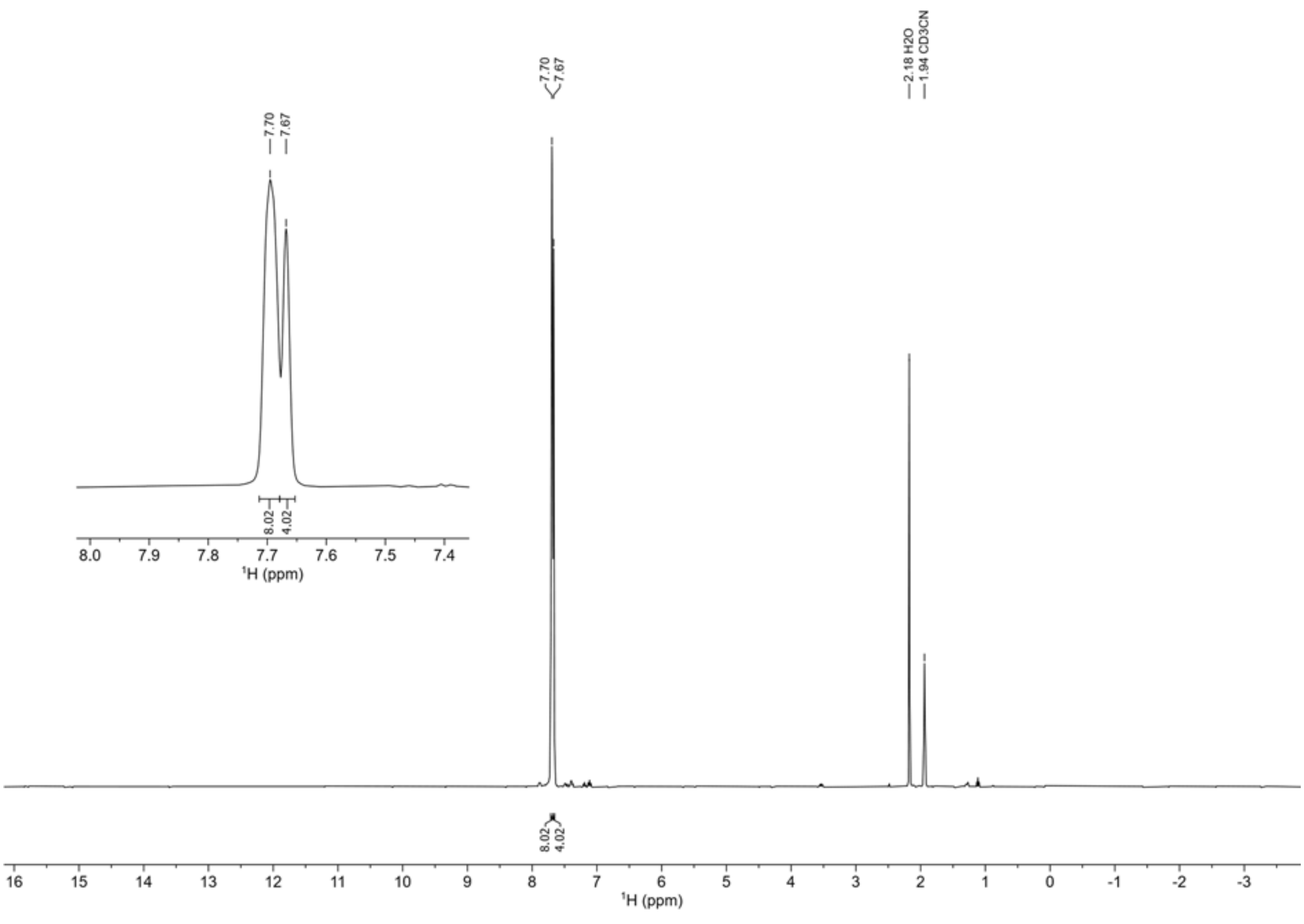

Figure S1 ^{1}H NMR spectrum of Tl(BAr^F) in CD_3CN solution at 295 K. The HDO peak arises from non-dried solvent. The spectrum shows no signs of solvent residues, but only peaks attributable to the BAr^F anion: 7.71-7.68 ppm (multiplet, 8H, *ortho*-H), 7.67 (singlet, 4H, *para*-H).

Remarks on Tl···F interactions in solution and NMR studies

To identify potential interactions in solution, akin to the solid-state structure, between the thallium cation and the CF_3 moieties of the BAr^F anion, we performed ^{19}F NMR experiments. We tested d_3-acetonitrile (see Figure S2) and fluorobenzene (see Figure S3) as solvents with differing coordination ability. This should essentially lead to a solvent-separated ion pair in acetonitrile solution and a (closer) contact ion pair in the less coordinating fluorobenzene. In both solvents, there is no sign of interaction at room temperature, since the CF_3 groups give rise to a sharp and clear singlet at ca. –63 ppm in both spectra.

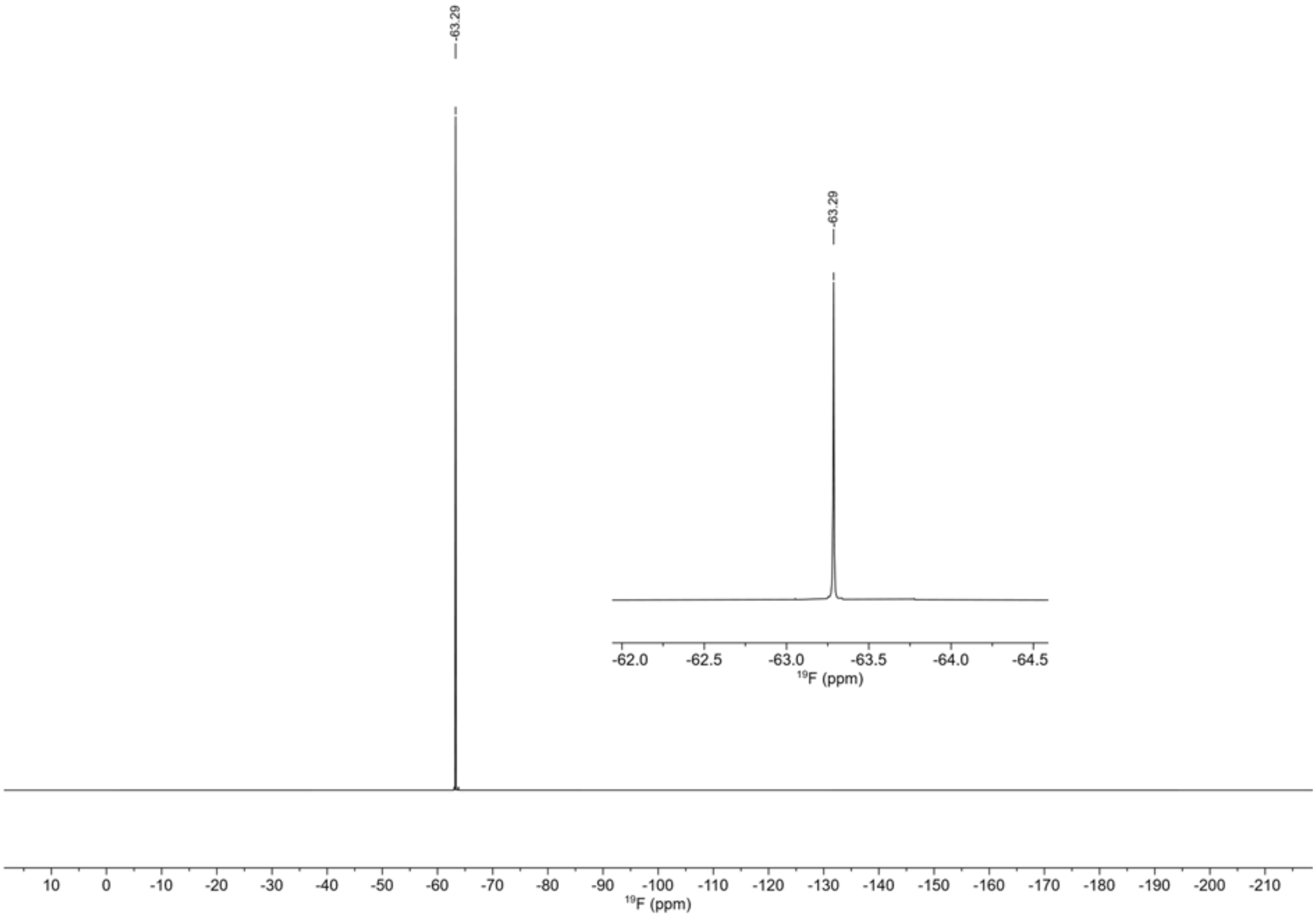

Figure S2 ^{19}F NMR spectrum of $Tl(BAr^F)$ in CD_3CN solution at 295 K. The spectrum and the zoom-in show a clean singlet at –63.29 ppm and therefore no signs of interaction between the CF_3 moieties of the anion and the Tl cation.

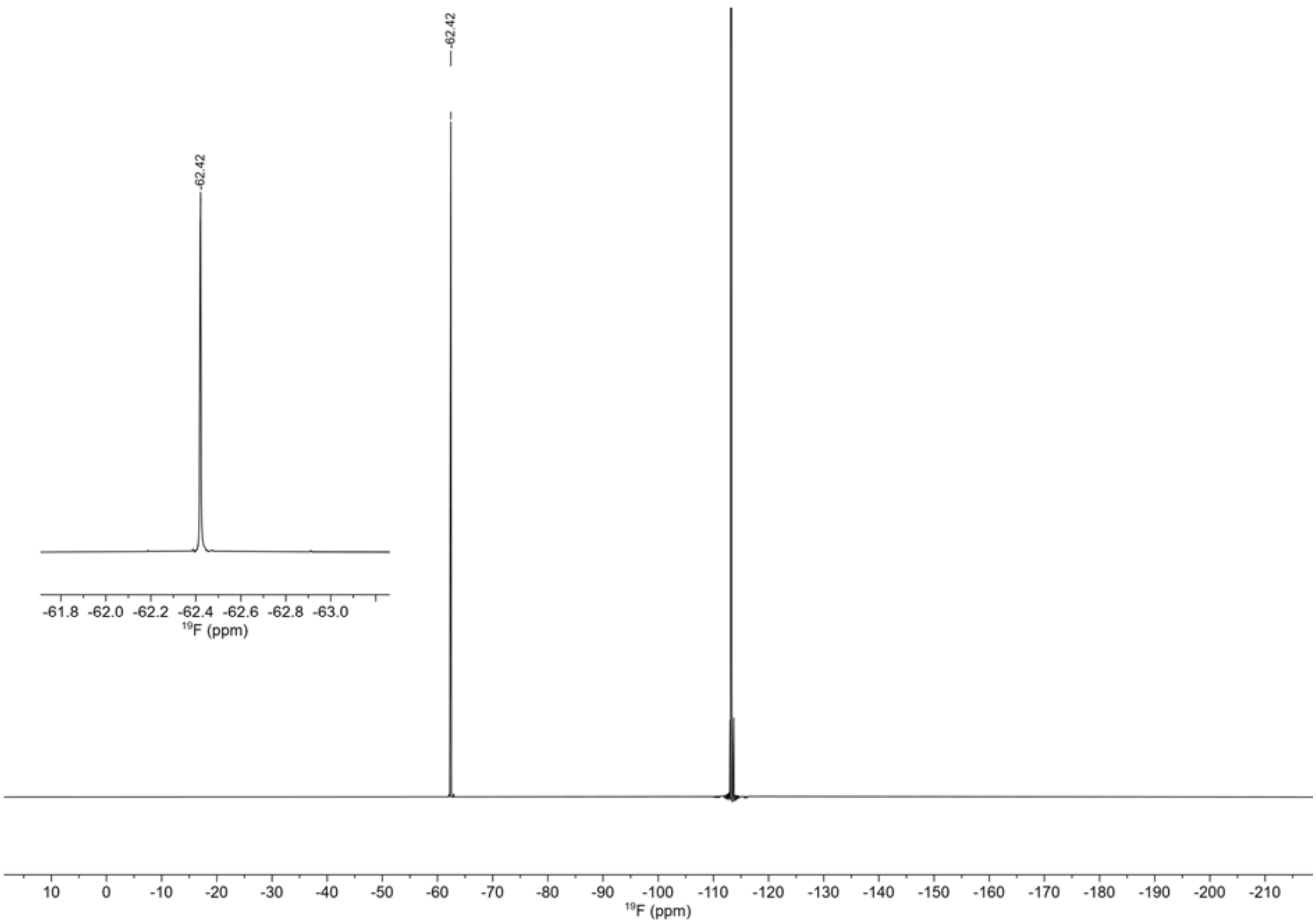

Figure S3 ^{19}F NMR spectrum of Tl(BAr^F) in fluorobenzene solution with 10 vol% C_6D_6 at 295 K. The spectrum and the zoom-in show a clean singlet at –62.42 ppm and therefore no signs of interaction between the CF_3 moieties of the anion and the Tl cation.

S2. Additional SC-XRD data

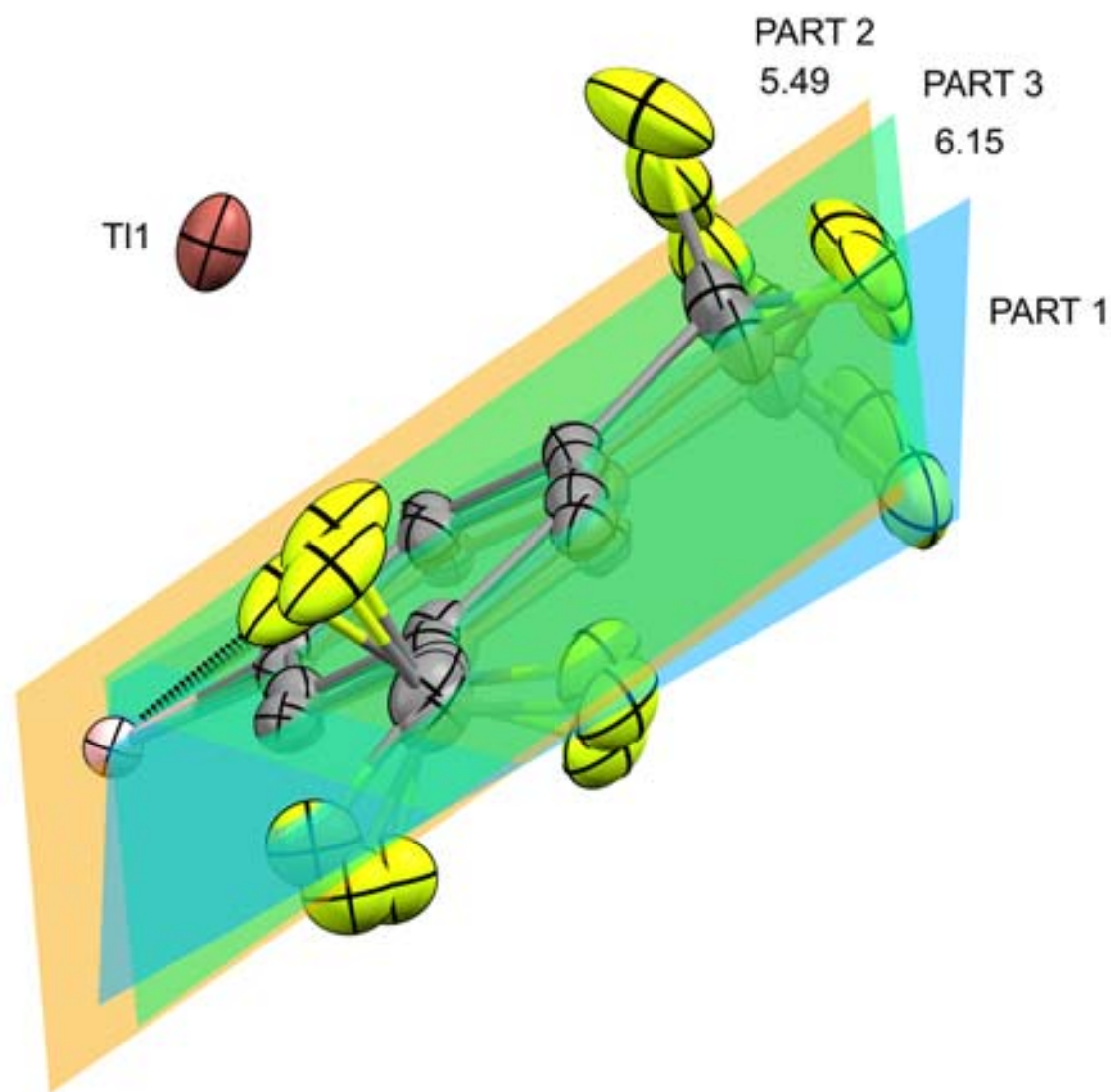

Figure S5 Disordered arene rings of the BAr^F anion in the asymmetric unit due to differing supramolecular interactions within the individual components of the disorder. Relative to PART 1, the individual ring planes tilt by 5.49° in PART 2 and by 6.15° in PART 3.

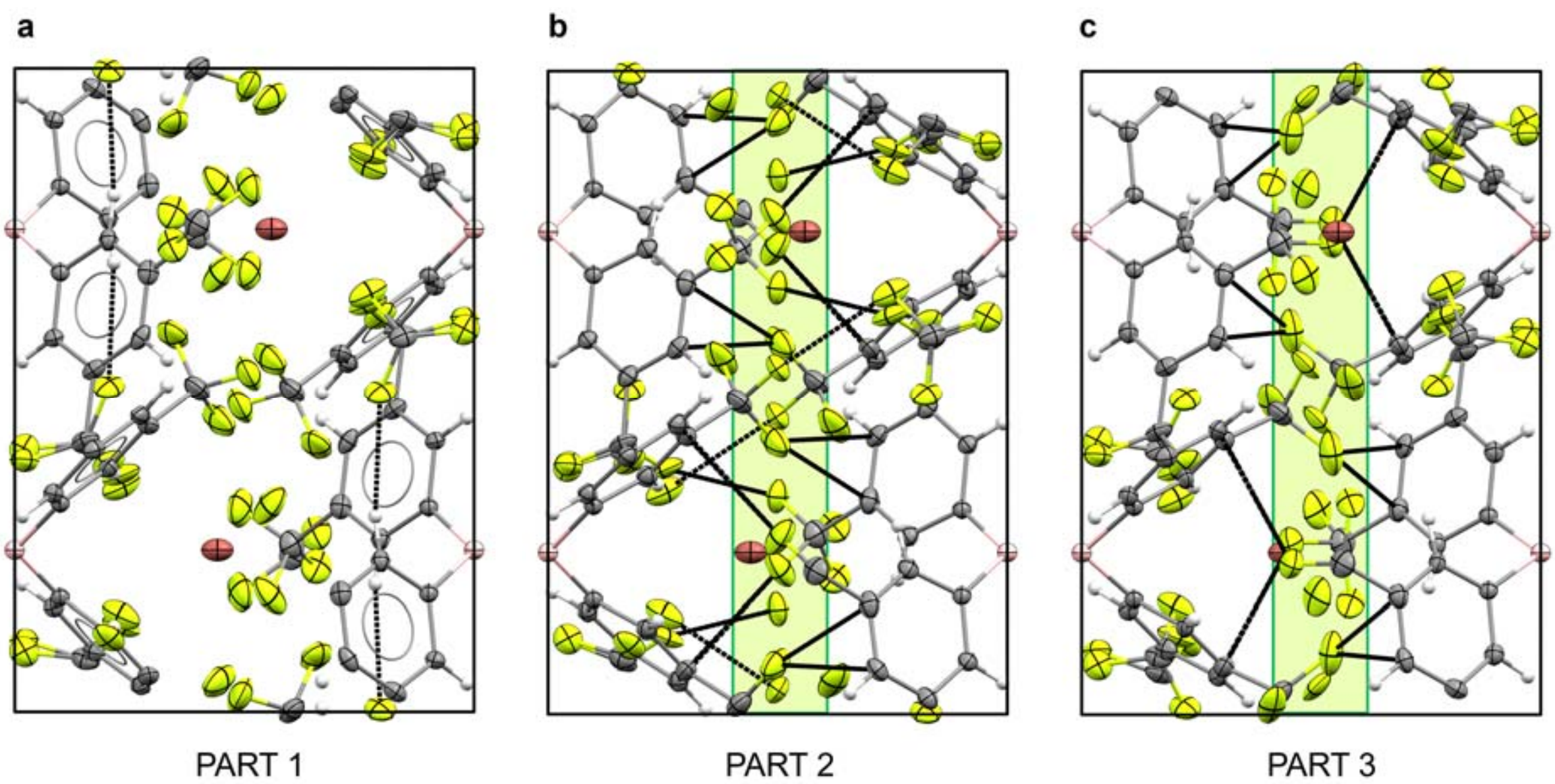

Figure S6 Layer-like structures in the packing of Tl(BArF), with (**a**) the major component of the disordered arene rings, and (**b**) and (**c**) as the minor components, as viewed along the *b*-axis. In (**a**), non-classical hydrogen bonds lead to layers parallel to the *a*- and *b*-axes, while in (**b**) and (**c**), a "fluorous" interlayer with short C–F···π and F···F contacts (both highlighted for easier visibility by a light-green box) forms parallel to the *a*- and *b*-axes and close to the thallium atoms.

Table S2 Refined third-order Gram-Charlier coefficients for Tl1, with standard uncertainties in parentheses, including their statistical significance (x/σ).

Coefficient	Value	x/σ	Significance
C111	–0.0	-	not significant
C112	–0.0	-	not significant
C113	$-1.23(9) \times 10^{-7}$	1.4	not significant
C122	–0.0	-	not significant
C123	–0.0	-	not significant
C133	–0.0	-	not significant
C222	–0.0	-	not significant
C223	$-1.23(9) \times 10^{-7}$	1.4	not significant
C233	–0.0	-	not significant
C333	$-4.11(8) \times 10^{-6}$	51.4	highly significant

The significance level was calculated as the absolute value of the refined coefficient divided by its standard uncertainty (x/σ). Coefficients with $x/\sigma < 3$ are considered not significant, those with $3 \leq x/\sigma < 5$ marginally significant, those with $5 \leq x/\sigma < 10$ significant, and those with $x/\sigma \geq 10$ highly significant.

Table S3 Refined fourth-order Gram-Charlier coefficients for Tl1, with standard uncertainties in parentheses, including their statistical significance (x/σ).

Coefficient	Value	x/σ	Significance
D1111	$-5.3(8) \times 10^{-9}$	6.6	significant
D1112	$6(2) \times 10^{-10}$	3.0	marginally significant
D1113	–0.0	-	not significant
D1122	$-9(3) \times 10^{-10}$	3.0	marginally significant
D1123	–0.0	-	not significant
D1133	$3.5(7) \times 10^{-9}$	5.0	significant
D1222	$-6(2) \times 10^{-10}$	3.0	marginally significant
D1223	–0.0	-	not significant
D1233	–0.0	-	not significant
D1333	–0.0	-	not significant

D2222	$-5.3(8) \times 10^{-9}$	6.6	significant
D2233	$3.5(7) \times 10^{-9}$	5.0	significant
D2333	–0.0	-	not significant
D3333	$4.20(11) \times 10^{-7}$	38.2	highly significant

The significance level was calculated as the absolute value of the refined coefficient divided by its standard uncertainty (x/σ). Coefficients with $x/\sigma < 3$ are considered not significant, those with $3 \leq x/\sigma < 5$ marginally significant, those with $5 \leq x/\sigma < 10$ significant, and those with $x/\sigma \geq 10$ highly significant.

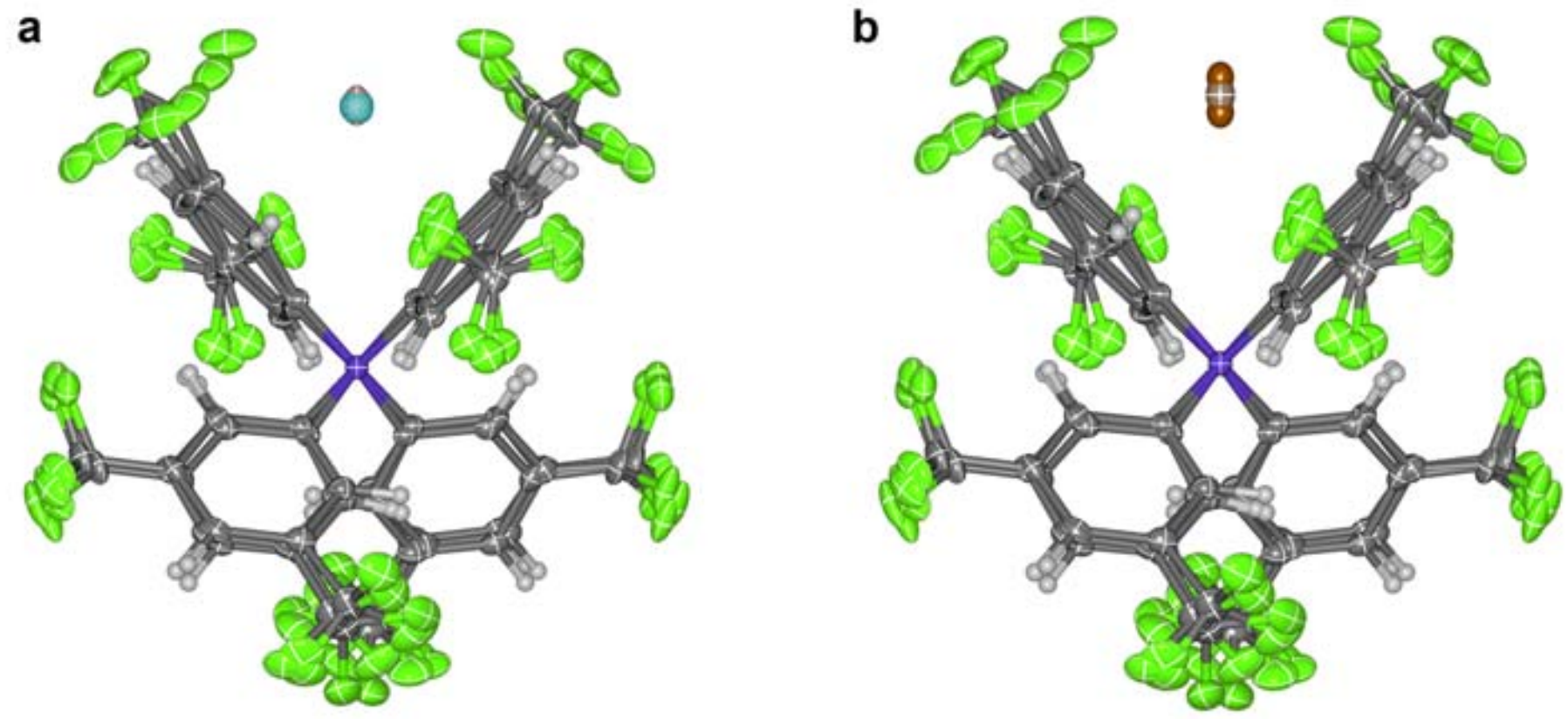

Figure S7 Visualisation of the results of fourth-order anharmonic refinement on Tl1, as viewed along the *a*-axis. (**a**) The probability distribution function (PDF) around Tl1 is positive (turquoise: positive; red: negative) and therefore physically meaningful. (**b**) Depiction of the anharmonic motion of Tl1 at a scale of 1.0. The direction of displacement lies directly on the fourfold axis, which is parallel to the *c*-axis.

S3. PXRD data

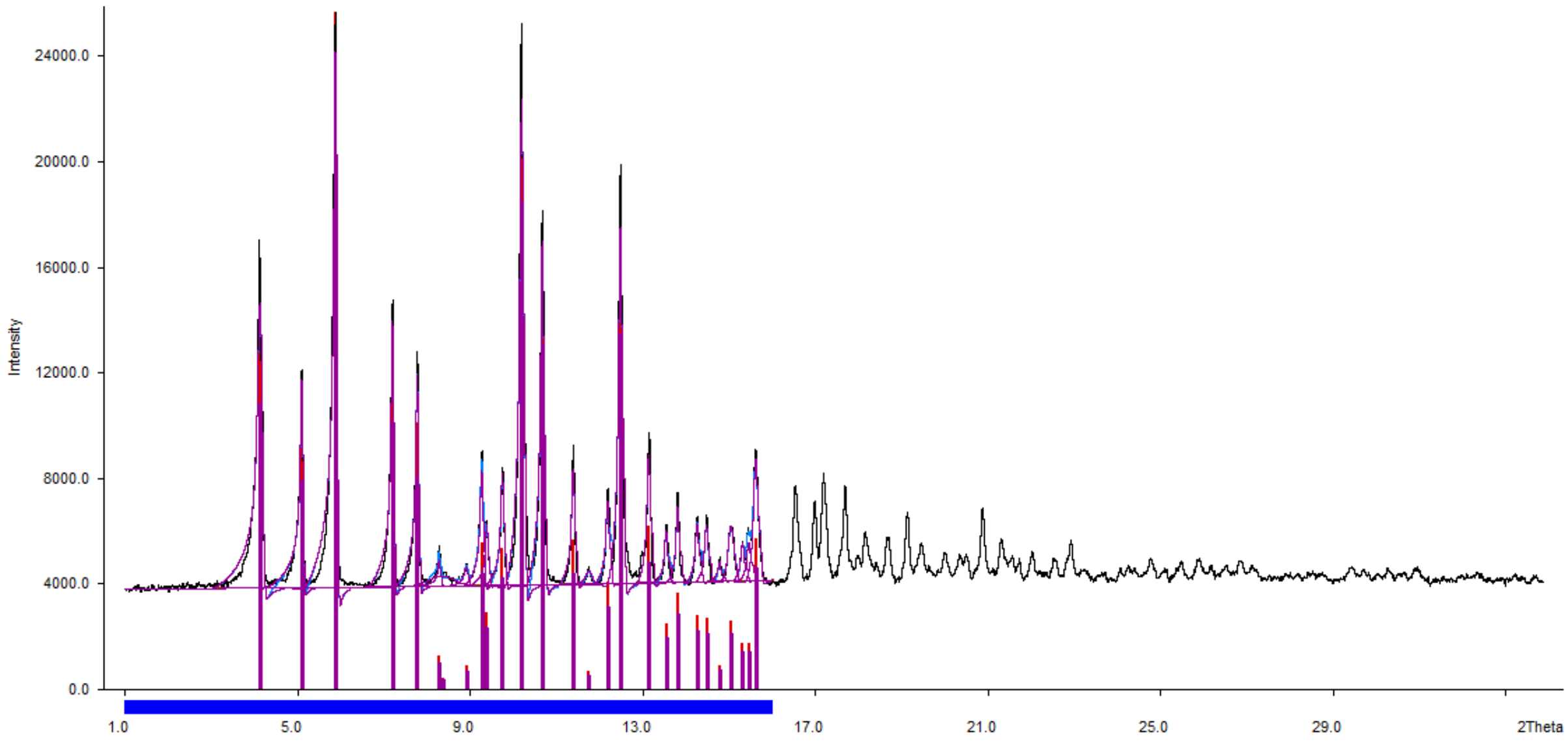

Figure S8 Overlay of the experimentally determined powder diffractogram at room temperature and the corresponding Pawley fit based on the simulated pattern at 100 K in the range of 2θ = 1-16°, with the experimental pattern (black), the experimental peaks (red bars), the simulated pattern (blue) and the simulated peaks (purple bars).
